# Deep learning with interactive segmentation for risk stratification of cystic renal lesions on tri-phase CT: a multicenter study

**DOI:** 10.3389/fonc.2026.1832969

**Published:** 2026-10-02

**Authors:** Wenlong Yuan, Pinglin He, Quanhao He, Xuan Zhang, Qing Jiang, Siwen Yin, Fajin Lv, Jie Liu, Weiyang He, Mingzhao Xiao

**Affiliations:** 1 Department of Urology, The First Affiliated Hospital of Chongqing Medical University, Chongqing, China; 2 Department of Urology, Affiliated Hospital of Chengdu University, Chengdu, Sichuan, China; 3 Department of Urology, Yongchuan Hospital of Chongqing Medical University, Chongqing, China; 4 Department of Urology, The Second Affiliated Hospital of Chongqing Medical University, Chongqing, China; 5 Department of Urology, Chongqing University Fuling Hospital, Chongqing, China; 6 Department of Radiology, The First Affiliated Hospital of Chongqing Medical University, Chongqing, China; 7 College of Artificial Intelligence Medicine, Chongqing Medical University, Chongqing, China

**Keywords:** Bosniak classification version 2019, computed tomography, cystic renal lesions, deep learning, interactive segmentation

## Abstract

**Background:**

Accurate preoperative risk stratification of cystic renal lesions (CRLs) remains a clinical challenge, primarily due to morphological overlap which often leads to the overtreatment of indolent cysts. To address this, we developed and validated an integrated deep learning framework that combines interactive segmentation with tri-phase CT feature integration to refine malignancy prediction.

**Methods:**

This retrospective multicenter study enrolled 434 patients with CRLs from four institutions, partitioned into training (n=195), validation (n=100), and external test (n=139) cohorts. The reference standard was histopathological diagnosis or ≥4 years of radiological stability. For volumetric segmentation, we implemented an interactive ‘human-in-the-loop’ Swin UNETR model. To integrate complementary information across the three CT phases, we then deployed a Tri-Phase-Channel (TPC) Perceiver architecture to integrate features extracted by parallel SEResNet50 backbones from unenhanced, corticomedullary, and nephrographic CT phases. Diagnostic performance was evaluated against the single-phase SEResNet50 models and the Bosniak classification version 2019 using the Area Under the Receiver Operating Characteristic Curve (AUC) and Decision Curve Analysis (DCA).

**Results:**

In the external test cohort, the interactive segmentation model yielded a Dice score of 0.890. For malignancy prediction, the TPC-Perceiver attained an AUC of 0.969 (95% CI: 0.911–1.000), significantly outperforming the Bosniak classification version 2019 (AUC 0.884; *p =0.016*). At the fixed probability threshold of 0.5, the model achieved a sensitivity of 94.4% and a specificity of 96.7%, compared with 100.0% and 76.9%, respectively, for the clinical standard. Furthermore, decision curve analysis indicated that the deep learning framework conferred greater net benefit across a broad range of threshold probabilities.

**Conclusion:**

The integrated framework showed favorable performance for cystic renal lesion risk stratification. Exploratory pairwise AUC comparisons favored the TPC Perceiver over the single-phase models and the clinical Bosniak v2019 assessment, while its AUC was comparable to that of the previous SETD model. The sensitivity-specificity profile observed at the fixed threshold suggests potential value in reducing false-positive risk stratification. Larger prospective multicenter studies are required before clinical implementation.

## 1 Introduction

The widespread adoption of cross-sectional imaging has led to a substantial increase in the incidental detection of cystic renal lesions (CRLs). These lesions are particularly prevalent in the aging population, with reported rates approaching 50% in individuals older than 50 years (1, 2). Although most CRLs are benign, approximately 20% of complex lesions are malignant (3). Preoperative differentiation is clinically difficult, primarily due to the morphological overlap between benign and malignant entities. Confounding features including septal thickness, nodular enhancement, and calcification often complicate the diagnosis, especially in early-stage lesions (1, 4).

The Bosniak classification remains the standard reference for radiological risk stratification. While the 2019 update (v2019) implemented stricter criteria to mitigate inter-reader variability, its practical utility has yielded mixed results. Dana et al. observed only marginal diagnostic improvements, noting a lack of significant gains in specificity for high-risk categories (III and IV) alongside persistent low sensitivity for equivocal cases (IIF and III) (5). Moreover, despite the intent to standardize terminology, inter-reader agreement for differentiating surgical (III–IV) from non surgical (I–IIF) candidates has actually declined in several validation studies. This discrepancy is often attributed to the difficulty in applying rigid quantitative thresholds to inherently heterogeneous morphological features (5–8).

This diagnostic uncertainty contributes to both overdiagnosis and overtreatment (9). A substantial proportion of surgically excised indeterminate cystic renal lesions are ultimately found to be benign, exposing some patients to interventions that may not provide oncologic benefits (10, 11). Such interventions incur healthcare costs and may expose patients to perioperative complications and long-term renal functional impairment (12). Conversely, underdiagnosis may delay the treatment of malignant lesions. Thus, there is a critical need to refine preoperative risk stratification while balancing oncologic safety and organ preservation.

To mitigate inter-reader variability, deep learning (DL) has emerged as a powerful approach in medical image analysis. Recent studies have demonstrated the broad applicability of DL to automated medical image segmentation and image-based diagnostic analysis, including hybrid segmentation strategies, U-Net-based delineation, CT-based disease detection, and AI-assisted analysis across different imaging modalities (13–16). In renal imaging specifically, radiomics and machine-learning approaches have shown promising performance for renal mass characterization and cystic renal lesion risk stratification (17–21). Compared with conventional radiomics pipelines based on predefined handcrafted descriptors, DL enables hierarchical, task-specific representations to be learned directly from imaging data, providing a flexible framework for characterizing complex imaging phenotypes.

Building upon our previous development of an interactive 3D U-Net and SETD classifier, we identified a critical limitation: the exclusion of the unenhanced phase. In standard renal protocols, malignancy is often defined by a net enhancement of ≥15 HU, a calculation that mandates baseline unenhanced attenuation (22). To address this gap, this multicenter study introduces a novel Interactive Multi-phase Framework. We employ an interactive Swin UNETR for robust segmentation across unenhanced, corticomedullary, and nephrographic phases, leverage SEResNet50 for phase-specific feature extraction, and integrate these representations via a Tri-Phase-Channel (TPC) Perceiver model. The objective of this study is to determine whether this multi-phase fusion architecture yields superior risk stratification for CRLs compared to existing methods.

## 2 Methods

### 2.1 Study participants and inclusion criteria

This retrospective, multicenter study was approved by the Institutional Review Board (IRB) of each participating center, with the requirement for informed consent waived. The study protocol strictly adhered to the CLEAR checklist guidelines (23). Cases were retrieved from the Picture Archiving and Communication Systems (PACS) using keywords related to cystic renal lesions (e.g., ‘Bosniak’, ‘complex renal cyst’), as detailed in **Figure 1**. The inclusion criteria were as follows: (a) a cystic renal lesion (CRL) with a solid component comprising <25% of the total lesion volume; (b) lesion diameter >1 cm; (c) availability of complete tri-phase CT examinations, including the unenhanced, corticomedullary, and nephrographic phases; and (d) availability of an eligible reference standard. Patients with incomplete multiphase CT examinations, prior renal surgery, or hereditary syndromes associated with renal lesions, including von Hippel–Lindau syndrome and polycystic kidney disease, were excluded. All included CT examinations were of sufficient quality for lesion evaluation and segmentation, and no cases were excluded solely because of image quality.

**Figure 1:**
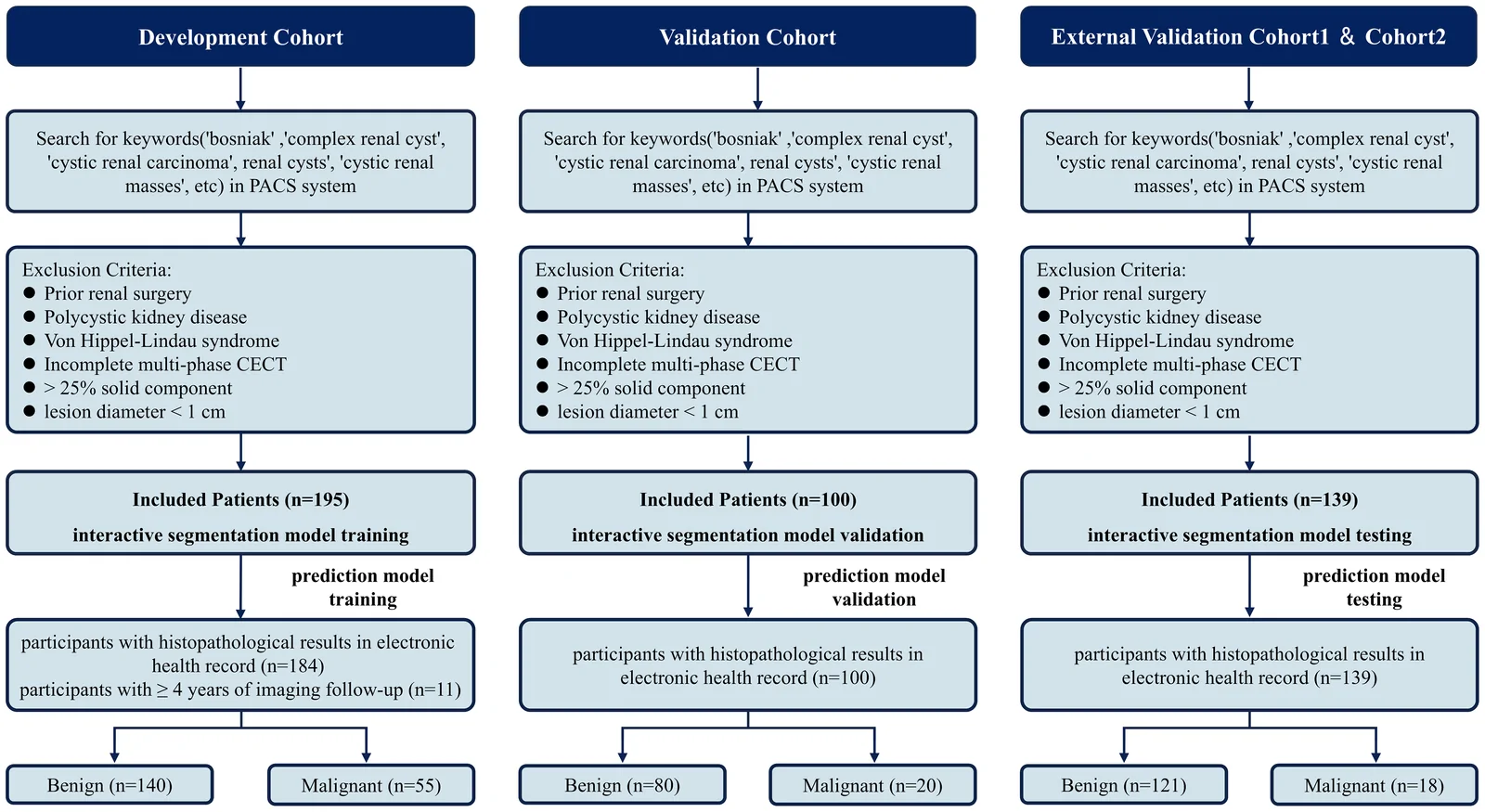
Flowchart of recruitment pathways, histopathological results, and follow-up outcomes for cystic renal lesions (CRLs). The validation cohort was employed for model validation, while external validation cohorts 1 and 2 were combined for model testing. PACS, Picture Archiving and Communication System; CECT, Contrast-Enhanced Computed Tomography.

Participants were allocated into three independent cohorts: a training cohort (n=195), an internal validation cohort (n=100), and a multicenter external validation cohort (combining two external centers, n=139). The two external validation cohorts were pooled to form a single multicenter external test cohort because the total number of malignant cases in the external dataset was limited (n=18). Separate center-specific evaluation would therefore have resulted in very small malignant subgroups and unstable diagnostic performance estimates. Pooling the two centers enabled a more stable overall estimate of external performance while retaining heterogeneity in scanner platforms and acquisition settings. Histopathological diagnosis served as the primary reference standard. In the training cohort, 184 cases were histopathologically confirmed, whereas 11 benign lesions without surgical pathology were included based on at least 4 years of CT follow-up demonstrating no definite imaging progression, including no upgrade in Bosniak classification. All cases in the internal validation and external test cohorts were histopathologically confirmed. Detailed recruitment pathways, histopathological results, and follow-up outcomes are illustrated in **Figure 1**.

### 2.2 Multi-institutional data acquisition

To ensure dataset diversity, imaging data were retrospectively collected from four independent institutions. The training cohort (The First Affiliated Hospital of Chongqing Medical University), validation cohort (The Second Affiliated Hospital of Chongqing Medical University), and external validation cohort 1 (Fuling Affiliated Hospital of Chongqing University) used 64-slice or 128-slice CT scanners for contrast-enhanced CT acquisition, while external validation cohort 2 (Yongchuan Affiliated Hospital of Chongqing Medical University) used a 256-slice spiral CT scanner. CT examinations were acquired using institution-specific renal CT protocols, as detailed in **Supplementary Table 2**. We expanded upon previous methodologies limited to corticomedullary and nephrographic phases (4) by additionally integrating the unenhanced phase. This tri phase strategy was adopted to enable the deep learning architecture to extract a richer, more comprehensive feature set.

### 2.3 Image preprocessing and standardization

To reduce variability associated with heterogeneous CT acquisition protocols across participating institutions, all lesion-centered CT volumes underwent the same preprocessing pipeline before model training and inference. Prior to feature extraction, 3D inter-phase image registration was performed using SimpleITK. Rigid followed by affine registration based on Mattes Mutual Information (50 histogram bins) was used to align the unenhanced (UP) and corticomedullary (CP) volumes to the nephrographic (NP) reference volume to reduce inter-phase respiratory misalignment. CT attenuation values were clipped to a fixed range of 0–250 HU using ScaleIntensityRanged. The resulting three-dimensional volumes were then resized to a fixed input matrix of 96 × 96 × 14 voxels using trilinear interpolation. During model training, random spatial augmentations, including rotation and affine transformations with translation and scaling, were applied to increase training variability. The validation and external test images underwent the same deterministic registration, intensity-clipping, and resizing procedures without random augmentation. No statistical batch-effect harmonization method, such as ComBat, was applied.

### 2.4 Clinical Bosniak v2019 assessment

The Bosniak classification version 2019 categories used as the clinical imaging comparator were retrospectively extracted from the finalized clinical radiology reports. In routine clinical practice at the participating institutions, contrast-enhanced CT examinations were interpreted through the standard departmental reporting workflow and underwent senior radiologist review before report finalization. Accordingly, the Bosniak categories used in the present analysis represent the finalized clinical radiology assessments, and individual pre-finalization ratings were not separately retained in the retrospective research dataset.

### 2.5 ROI annotation

Two radiologists (>10 years of clinical experience) participated in the delineation and review of the regions of interest (ROIs) of CRLs using ITK-SNAP software. To ensure segmentation precision, the readers cross-referenced multi-planar (axial, sagittal, and coronal) reformations across the unenhanced, corticomedullary, and nephrographic phases. When disagreement regarding lesion boundaries occurred, a senior radiologist (>20 years of clinical experience) participated in the review to establish the final reference annotation. Quantitative interobserver variability of the initial segmentation masks was not separately assessed.

### 2.6 Interactive swin UNETR architecture and segmentation workflow

The overall workflow of the proposed AI system is illustrated in **Figure 2**. In this study, we employed the Swin UNETR architecture, which combines a hierarchical 3D Swin Transformer for global context extraction with a CNN-based decoder via skip connections. By fusing global representations with local spatial details, this hybrid framework captures multi-scale information. To enable interactive segmentation refinement, we integrated a user-interaction module that enables clinicians to refine segmentation masks via positive and negative guidance points (see **Supplementary Figure 1**).

**Figure 2:**
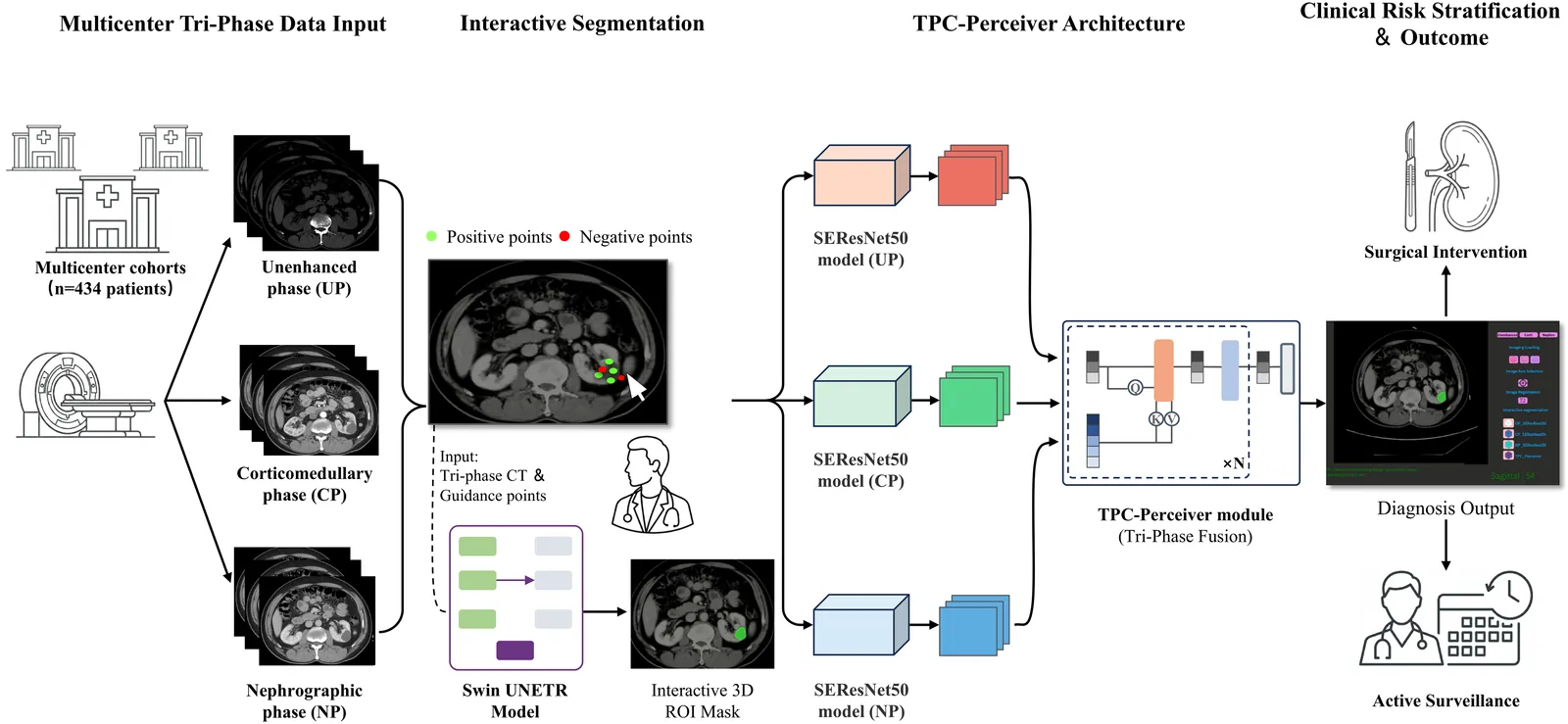
Overall workflow illustrating the detailed procedures for constructing the cystic renal lesions (CRLs) artificial intelligence (AI) system.

During model training, interaction prompts were algorithmically simulated to train the network to respond to corrective inputs. During evaluation of the validation and external test cohorts, positive and negative interaction points were manually provided by the radiologists based on the current segmentation output. The interaction procedure consisted of an initialization stage for coarse lesion localization followed by iterative refinement to correct residual under- or over-segmentation. Positive interaction points indicated lesion regions requiring inclusion, whereas negative interaction points indicated falsely included background or adjacent structures requiring exclusion. After each interaction, the segmentation mask was automatically updated by the model. No fixed upper limit was imposed on the number of interactions. Refinement was terminated when visual inspection of the three-dimensional segmentation indicated satisfactory lesion boundary delineation and the radiologist considered that no further correction was necessary.

### 2.7 Phase-specific feature extraction using SEResNet50

The unenhanced (UP), corticomedullary (CP), and nephrographic (NP) CT volumes were processed independently using three phase-specific 3D SEResNet50 networks. The SEResNet50 backbone incorporates Squeeze-and-Excitation (SE) modules within the residual architecture to perform adaptive channel-wise feature recalibration. Each network generated a high-level feature representation of its corresponding CT phase, which was subsequently provided to the TPC-Perceiver fusion module.

### 2.8 Tri-phase-channel perceiver fusion architecture

The Tri-Phase-Channel (TPC)-Perceiver was designed to integrate the high-level feature representations independently extracted from the unenhanced (UP), corticomedullary (CP), and nephrographic (NP) CT phases. The three phase-specific representations were provided to a latent-bottleneck fusion module, in which a fixed set of learnable latent queries aggregated information from the phase inputs through cross-attention. The latent representations were subsequently refined through attention-based processing, allowing complementary phase-specific information to interact within a shared latent space. The final fused representation was then used to generate the probability of malignancy.

The Perceiver architecture was selected because its latent-bottleneck and cross-attention formulation provides a structured mechanism for integrating heterogeneous phase-specific representations rather than relying solely on direct concatenation or averaging. A detailed schematic of the architecture is provided in **Supplementary Figure 2**, and implementation details and network configurations are available in the publicly accessible source-code repository.

### 2.9 SETD comparator

The previously developed Spatial Encoder Temporal Decoder (SETD) model was included as a comparative deep-learning baseline (4). Briefly, SETD employs a pretrained 3D-ResNet50-based spatial encoder to extract spatial features from multiphase CT images, followed by a two-layer gated recurrent unit (GRU)-based temporal decoder to model feature changes across CT phases. The GRU output is subsequently passed through a linear layer to generate the probability of malignancy. The original SETD model was developed using corticomedullary and nephrographic phase CT images, whereas the present TPC-Perceiver framework additionally incorporates the unenhanced phase.

### 2.10 Statistical analysis

Segmentation fidelity was evaluated using the Dice Similarity Coefficient (DSC), Intersection over Union (IoU), sensitivity, and specificity. Diagnostic performance was assessed using the area under the receiver operating characteristic curve (AUC), accuracy (ACC), sensitivity, specificity, positive predictive value (PPV), negative predictive value (NPV), and F1-score. For all deep-learning models, binary predictions and threshold-dependent performance metrics were calculated using a fixed probability threshold of 0.5 in both the internal validation and external test cohorts. Clinical utility was further assessed using decision curve analysis (DCA) to estimate the net benefit across threshold probabilities. Diagnostic performance of the TPC-Perceiver model was compared with the single-phase models, the previous SETD model, and the Bosniak classification version 2019. Differences in AUC were evaluated using the DeLong test. Statistical significance was defined as a two-sided *p* value < 0.05. No adjustment for multiple comparisons was applied; therefore, pairwise model comparisons were considered exploratory.

For accuracy, sensitivity, specificity, positive predictive value, and negative predictive value, 95% confidence intervals were calculated using the exact binomial (Clopper–Pearson) method. Confidence intervals for positive and negative likelihood ratios were calculated on the logarithmic scale. The 95% confidence intervals for AUC were estimated using the DeLong method. Because this was a retrospective study, the size of the external test cohort was determined by the number of eligible cases available during the predefined study period. Therefore, the precision of the external test estimates was evaluated from the observed sample size and corresponding 95% confidence intervals.

### 2.11 Model training and class imbalance handling

To mitigate class imbalance in the training cohort (140 benign and 55 malignant lesions), the classification models were trained using a class-weighted cross-entropy loss. Class-specific weights were calculated as wc​=1/log(1.02+fc​), where fc denotes the relative frequency of class c in the training set, thereby assigning a greater loss contribution to the minority malignant class. No oversampling, undersampling, focal loss, or synthetic resampling was applied. The validation and external test cohorts were retained in their original class distributions during performance evaluation.

### 2.12 Computational hardware and software

All experiments were implemented in Python. PyTorch 2.0.0 was used as the primary deep learning framework and MONAI 1.3 was used for medical image loading, preprocessing, and augmentation (including LoadImaged, RandAffined, and ScaleIntensityRanged). The Perceiver implementation used the perceiver-pytorch library. Data management and statistical analysis were performed using NumPy 1.24.1 and Pandas 2.1.2, while Scikit-learn was used to calculate performance metrics including AUC, sensitivity, and specificity. Confusion matrices and ROC curves, were generated using Matplotlib and Seaborn. Model training and inference were accelerated using an NVIDIA A6000 GPU with 48 GB of memory. Code for the interactive segmentation model, TPC-Perceiver model, and data processing procedures is publicly available on GitHub (https://github.com/yuanwl2000/CRLs_AI).

## 3 Results

### 3.1 Patient clinical characteristics

The patient characteristics of each cohort are summarized in **Table 1**. We included a total of 434 patients from 2012 to 2025, divided into a training cohort (n=195), an internal validation cohort (n=100), and a multicenter external validation cohort (n=139) serving as the test cohort. Based on the Bosniak classification version 2005, 11 CRLs in the training cohort were originally classified into categories II (n=8) and III (n=3). These CRLs were confirmed to be benign renal cysts by radiological stability over 4 years of follow-up. Notably, one category III lesion was reclassified as Bosniak IV according to the updated 2019 Bosniak criteria. Histopathological confirmation was obtained for all other lesions in the training cohort, as well as for all lesions in the internal and external validation cohorts.

**Table 1:** Patient demographics and baseline characteristics of the study cohorts.

Characteristics	Training cohort	Validation cohort	External validation cohort1 & cohort2
Patients, n	195	100	139
Age, years, (median-IQR)	57(48-63)	57.5(49-67)	60(51-70)
Sex, n			
Sex(male)	121	56	84
Sex(female)	74	44	55
Lesion Diagnosis, n			
Benign(n=341)	140	80	121
Malignant (n=93)	55	20	18
Benign Reference, n			
≥4 years of imaging follow-up	11	0	0
Histopathology	129	80	121
Institution Distribution, n			
The First Affiliated Hospital	195		
The Second Affiliated Hospital		100	
Fuling Affiliated Hospital			80
Yongchuan Affiliated Hospital			59

Data for age are median (interquartile range). In the training cohort, all imaging follow-up assessments of CRLs were conducted over a period of ≥ 4 years. All CRLs in the external validation cohorts had histopathological results. CRLs, cystic renal lesions; IQR, interquartile range.

### 3.2 Performance of the interactive segmentation framework

The interactive Swin UNETR model demonstrated robust segmentation fidelity across all datasets, surpassing our previous 3D R-net baseline. Specifically, the Swin UNETR achieved a mean Dice Similarity Coefficient (DSC) of 0.885 ± 0.100 in the validation cohort and 0.890 ± 0.120 in the test cohort (**Table 2**). These results represent a notable improvement over the 3D R-net (validation DSC: 0.847 ± 0.086; test DSC: 0.861 ± 0.072).

**Table 2:** Performance of segmentation models in validation and test datasets.

Model	DSC (%)	IoU (%)	SEN (%)	SPE (%)
Validation cohort	88.48 ± 9.95	80.53 ± 13.54	88.77 ± 9.64	99.98 ± 0.06
Test cohort	89.04 ± 12.03	81.73 ± 14.11	87.92 ± 9.96	99.99 ± 0.02

All performance metrics are reported as mean values along with their respective standard deviations. DSC, Dice Similarity Coefficient; SEN, Sensitivity; SPE, Specificity.

To further characterize the practical interaction burden of the human-in-the-loop workflow, the number of user clicks required during segmentation was recorded. During the initialization stage, the median number of clicks was 10 in both cohorts, with mean values of 9.83 and 9.84 in the validation and external test cohorts, respectively. During the subsequent refinement stage, the median number of corrective clicks was 6 in both cohorts, with corresponding mean values of 4.62 and 4.91. The distributions of initialization and refinement interactions are presented in **Supplementary Figure 4**.

**Figure 3** illustrates representative segmentation outputs, highlighting the model’s efficacy in refining boundaries via user-guided positive and negative clicks, particularly for lesions with complex or irregular morphologies.

**Figure 3:**
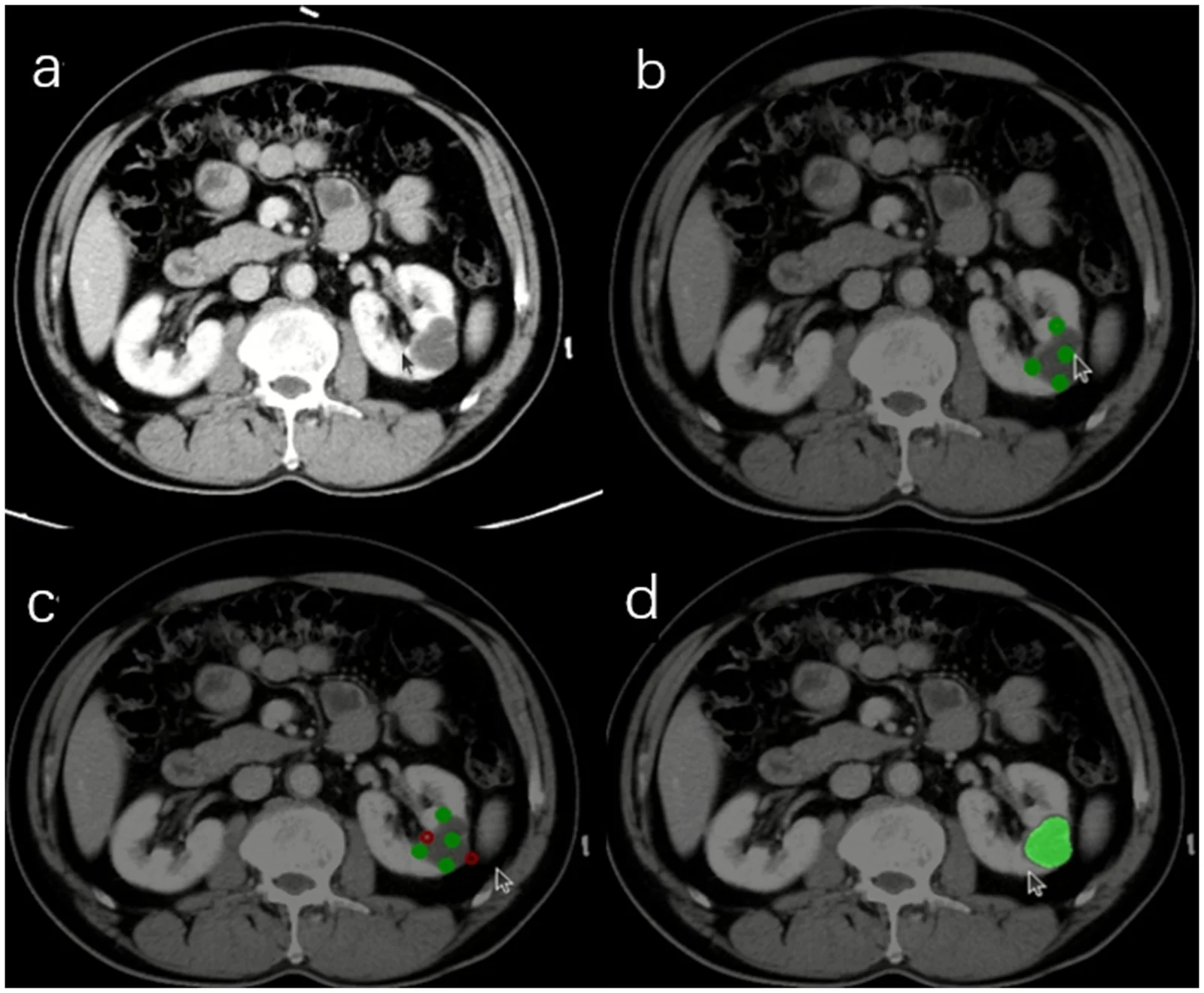
Example usage of the initial CRLs segmentation model and refined CRLs segmentation model. **(a)** Original image inputs, **(b)** initial segmentation results combined with positive indicator points (green dots), **(c)** initial segmentation results combined with negative indicator points (red dots), **(d)** refined segmentation results.

### 3.3 Diagnostic performance of the clinical Bosniak v2019 assessment

The Bosniak v2019 categories documented in the finalized clinical radiology reports were used as the clinical imaging comparator, reflecting the routine departmental reporting and senior-review workflow rather than a dedicated multi-reader research assessment. Lesions were grouped into benign (I, II) and potentially malignant (IIF, III, IV) categories to serve as the clinical baseline. Although this approach achieved 100% sensitivity in both the validation and test cohorts, it yielded limited specificity (77.5% and 76.9%, respectively), indicating a substantial rate of false-positive classifications. In the external test cohort, the clinical Bosniak assessment achieved an AUC of 0.884 (95% CI: 0.847–0.922), which was significantly lower than that of the TPC-Perceiver (DeLong *p* = 0.016). The corresponding diagnostic performance is summarized in **Table 3**, **Figure 4**.

**Table 3:** Diagnostic performance of each model and Bosniak classification version 2019 in the validation and test cohorts.

Cohort	Model	AUC (95% CI)	ACC (95% CI)	SEN	SPE	*p* value
Validation cohort	SETD	0.923 (0.872-0.974)	0.850 (0.765–0.914)	0.950 (0.751–0.999)	0.825 (0.724–0.901)	*p=0.155*
	SEResNet50_UP	0.946 (0.896-0.996)	0.910 (0.836–0.958)	0.850 (0.621–0.968)	0.925 (0.844–0.972)	*p=0.061*
	SEResNet50_CP	0.949 (0.898-1.000)	0.920 (0.848–0.965)	0.800 (0.563–0.943)	0.950 (0.877–0.986)	*p=0.063*
	SEResNet50_NP	0.889 (0.775-1.000)	0.910 (0.836–0.958)	0.650 (0.408–0.846)	0.975 (0.913–0.997)	*p=0.980*
	TPC-Perceiver	0.984 (0.965-1.000)	0.940 (0.874–0.978)	0.900 (0.683–0.988)	0.950 (0.877–0.986)	*p* < 0.001
	Bosniak classification version 2019	0.887 (0.841-0.934)	0.820 (0.731–0.890)	1.000 (0.832–1.000)	0.775 (0.668–0.861)	Reference
Test cohort	SETD	0.969 (0.941-0.997)	0.921 (0.863–0.960)	0.833 (0.586–0.964)	0.934 (0.874–0.971)	*p* < 0.001
	SEResNet50_UP	0.952 (0.907-0.997)	0.892 (0.828–0.938)	0.889 (0.653–0.986)	0.893 (0.823–0.942)	*p=0.020*
	SEResNet50_CP	0.937 (0.858-1.000)	0.942 (0.890–0.975)	0.778 (0.524–0.936)	0.967 (0.918–0.991)	*p=0.229*
	SEResNet50_NP	0.937 (0.876-0.998)	0.914 (0.854–0.955)	0.667 (0.410–0.867)	0.950 (0.895–0.982)	*p=0.143*
	TPC-Perceiver	0.969 (0.911-1.000)	0.964 (0.918–0.988)	0.944 (0.727–0.999)	0.967 (0.918–0.991)	*p=0.016*
	Bosniak classification version 2019	0.884 (0.847-0.922)	0.799 (0.723–0.861)	1.000 (0.815–1.000)	0.769 (0.683–0.840)	Reference

Accuracy, sensitivity, and specificity for all deep-learning models were calculated using a fixed probability threshold of 0.5 in both cohorts. ROC-AUC and DeLong comparisons were based on continuous prediction probabilities and were independent of this threshold. *p* values were derived from the DeLong test comparing the AUC of each model with that of the clinical Bosniak v2019 assessment (reference). Unenhanced phase [UP], Corticomedullary phase [CP], and Nephrographic phase [NP]; AUC, area under the receiver operating characteristic curve; ACC, accuracy score; 95% CI, 95% confidence interval.

**Figure 4:**
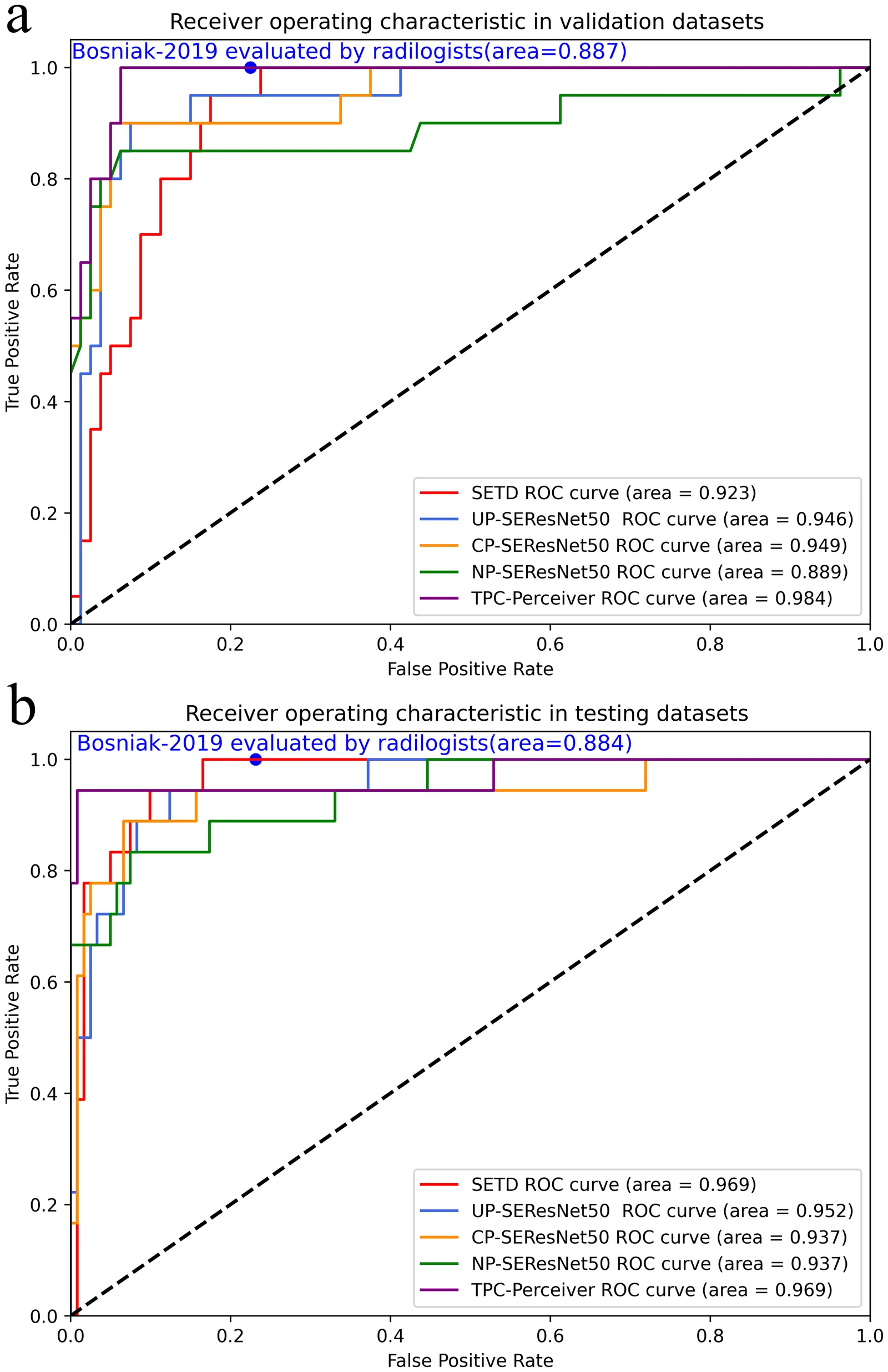
Receiver operating characteristic (ROC) curves for the models in **(a)** the validation datasets, **(b)** the testing datasets. UP, unenhanced phase; CP, corticomedullary phase; NP, nephrographic phase.

### 3.4 Performance of single-phase models (SEResNet50)

Among the single-phase models, CP-SEResNet50 achieved the highest AUC in the internal validation cohort (0.949), followed by UP-SEResNet50 (0.946) and NP-SEResNet50 (0.889). In the external test cohort, UP-SEResNet50 achieved the highest AUC (0.952), whereas CP-SEResNet50 and NP-SEResNet50 each achieved an AUC of 0.937. Detailed performance metrics are presented in **Table 3**.

### 3.5 Performance of TPC-perceiver model

The TPC-Perceiver demonstrated favorable diagnostic performance relative to the single-phase models, the previous SETD model, and the clinical Bosniak v2019 assessment. By integrating complementary multi-phase features extracted via phase-specific SEResNet50 backbones, the model yielded an AUC of 0.984 (95% CI: 0.965–1.000) in the validation cohort and 0.969 (95% CI: 0.911–1.000) in the test cohort. At the fixed probability threshold of 0.5, the model correctly classified 17 of 18 malignant lesions and 117 of 121 benign lesions in the external test cohort, corresponding to a sensitivity of 94.4% (95% CI, 72.7–99.9%) and a specificity of 96.7% (95% CI, 91.8–99.1%). The corresponding positive and negative likelihood ratios were 28.57 (95% CI, 10.83–75.38) and 0.057 (95% CI, 0.009–0.386), respectively. Exploratory pairwise DeLong comparisons yielded p-values of 0.047, 0.040, and 0.029 versus the UP, CP, and NP models, respectively, and 0.016 versus the clinical Bosniak v2019 assessment. No adjustment for multiple comparisons was applied. The AUC was comparable to that of the previous SETD model (p = 0.094); at the same fixed threshold, the TPC-Perceiver showed higher specificity (96.7% vs. 93.4%) and F1-score (0.872 vs. 0.732). The detailed performance metrics are presented in **Tables 3**, **4**.

**Table 4:** Additional diagnostic performance metrics of each model and Bosniak classification version 2019 in the test cohorts.

Cohort	Model	PPV (95% CI)	NPV (95% CI)	F1	*p* value
Test cohort	SETD	0.652 (0.427–0.836)	0.974 (0.926–0.995)	0.732	*p=0.094*
	SEResNet50_UP	0.552 (0.357–0.736)	0.982 (0.936–0.998)	0.681	*p=0.047*
	SEResNet50_CP	0.778 (0.524–0.936)	0.967 (0.918–0.991)	0.778	*p=0.040*
	SEResNet50_NP	0.667 (0.410–0.867)	0.950 (0.895–0.982)	0.667	*p=0.029*
	TPC-Perceiver	0.810 (0.581–0.946)	0.992 (0.954–1.000)	0.872	Reference
	Bosniak classification version 2019	0.391 (0.251–0.546)	1.000 (0.961–1.000)	0.562	*p=0.016*

PPV, NPV, and F1-score for all deep-learning models were calculated using the fixed probability threshold of 0.5. *p* values were derived from the DeLong test comparing the AUC of each model with that of the TPC-Perceiver (reference). Unenhanced phase [UP], Corticomedullary phase [CP], and Nephrographic phase [NP]; PPV, positive predictive value; NPV, negative predictive value; F1, F1-score; 95% CI, 95% confidence interval.

### 3.6 Ablation analysis of multi-phase fusion strategies

To further examine the contribution of the proposed feature-fusion architecture, we compared the TPC-Perceiver with four probability-level combination strategies based on the outputs of the same phase-specific SEResNet50 backbones: equal-probability averaging, logistic-regression stacking, engineered probability features followed by logistic regression, and confidence-weighted probability pooling. These comparators combined phase-specific prediction probabilities, whereas the TPC-Perceiver fused latent feature representations.

In the validation cohort, the TPC-Perceiver achieved an AUC of 0.984, compared with 0.975, 0.974, 0.975, and 0.979 for equal-probability averaging, logistic regression stacking, engineered probability features with logistic regression, and confidence-weighted probability pooling, respectively. In the external test cohort, the corresponding AUCs were 0.969 for the TPC-Perceiver and 0.983 for each probability-level comparator. Formal pairwise significance results are not reported here because a valid paired comparison requires verified patient-level alignment across prediction files. At the common fixed threshold of 0.5, the methods showed different sensitivity-specificity trade-offs: the TPC-Perceiver achieved an accuracy of 96.4%, sensitivity of 94.4%, specificity of 96.7%, and F1-score of 0.872. Across the probability-level comparators, accuracy ranged from 92.1% to 97.1%, sensitivity from 72.2% to 94.4%, specificity from 91.7% to 99.2%, and F1-score from 0.756 to 0.882 (**Supplementary Table 4**). No strategy was uniformly superior across all threshold dependent metrics.

### 3.7 Clinical utility and error analysis

The ROC curves in **Figure 4** show the discriminative performance of the models, and the radar charts in **Figure 5** summarize their threshold-dependent diagnostic profiles. Confusion-matrix analysis (**Figure 6**) further characterizes the errors of the TPC-Perceiver and SETD models at the fixed probability threshold of 0.5. In the test cohort, the TPC-Perceiver misclassified one malignant lesion and four benign lesions, whereas the Bosniak v2019 assessment produced more false-positive classifications. Visual inspection of the calibration curves suggested approximate agreement between predicted and observed risks, although formal calibration parameters were not estimated (**Figure 7**). Decision Curve Analysis (DCA) indicated that the TPC-Perceiver model demonstrated a higher estimated net benefit than the “treat-all” and “treat-none” strategies over portions of the evaluated threshold range in both cohorts (**Figure 8**). These retrospective findings suggest potential clinical utility, but prospective evaluation is required to determine whether model use would reduce unnecessary interventions while maintaining malignancy detection. **Supplementary Figure 3** shows a detailed confusion matrix for the single-phase models.

**Figure 5:**
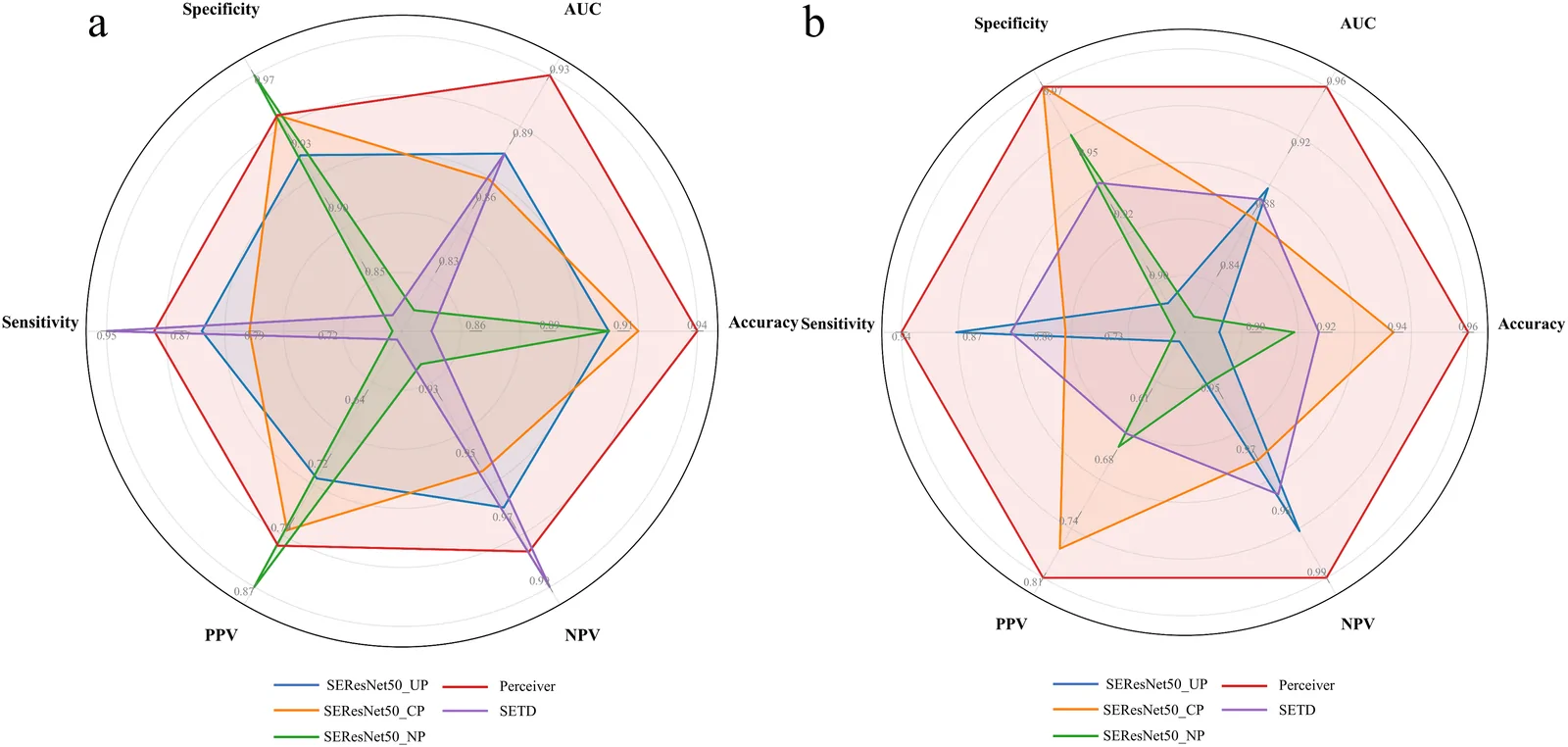
Radar charts summarizing the diagnostic performance of each model in **(a)** the validation cohort and **(b)** the external test cohort. UP, unenhanced phase (SEResNet50_UP); CP, corticomedullary phase (SEResNet50_CP); NP, nephrographic phase (SEResNet50_NP).

**Figure 6:**
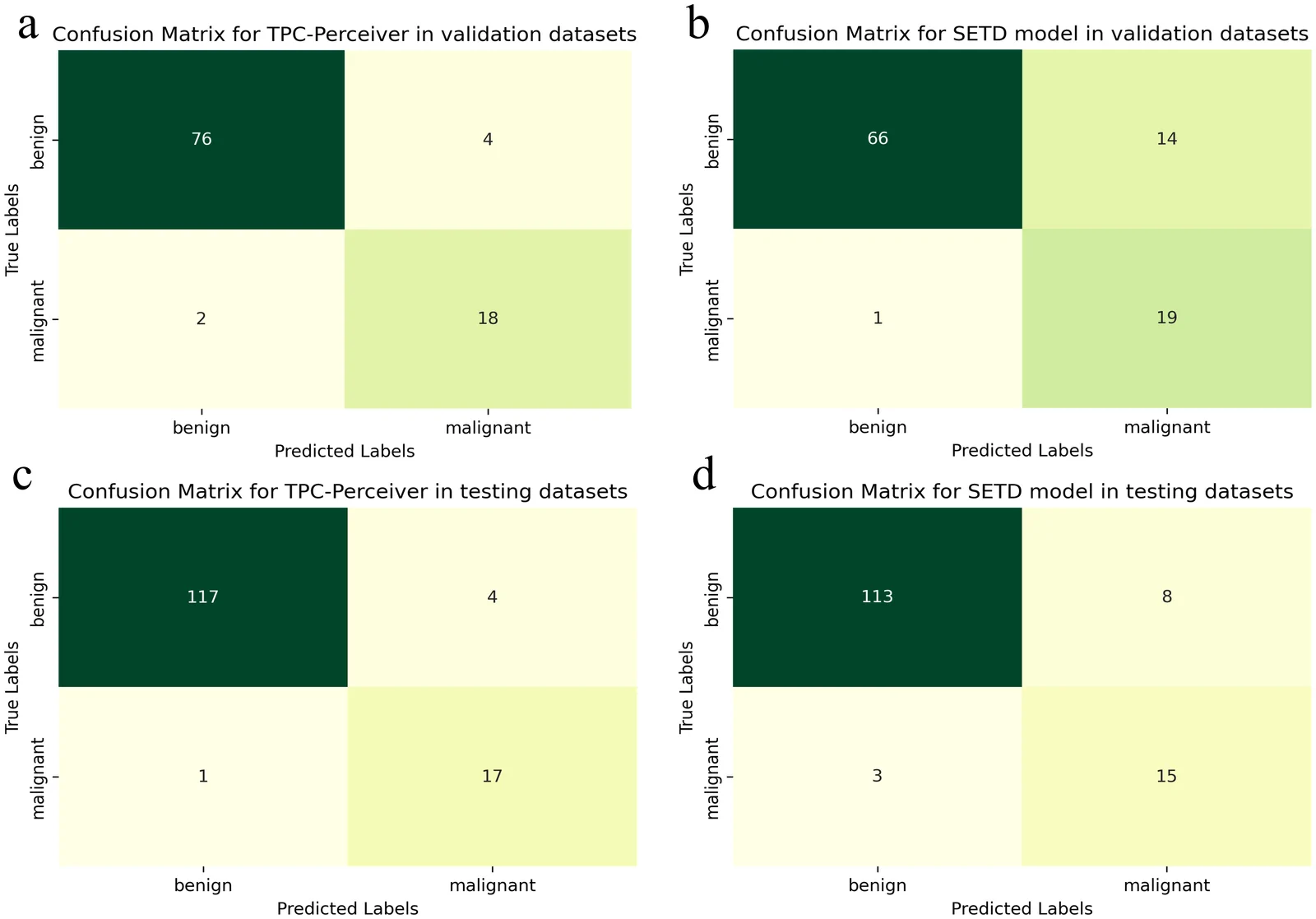
Confusion matrices for the TPC-Perceiver model and the SETD model in validation datasets and test datasets. **(a)** Confusion matrix for TPC-Perceiver model in validation datasets, **(b)** Confusion matrix for the SETD model in validation datasets, **(c)** Confusion matrix for TPC-Perceiver model in testing datasets, **(d)** Confusion matrix for the SETD model in testing datasets.

**Figure 7:**
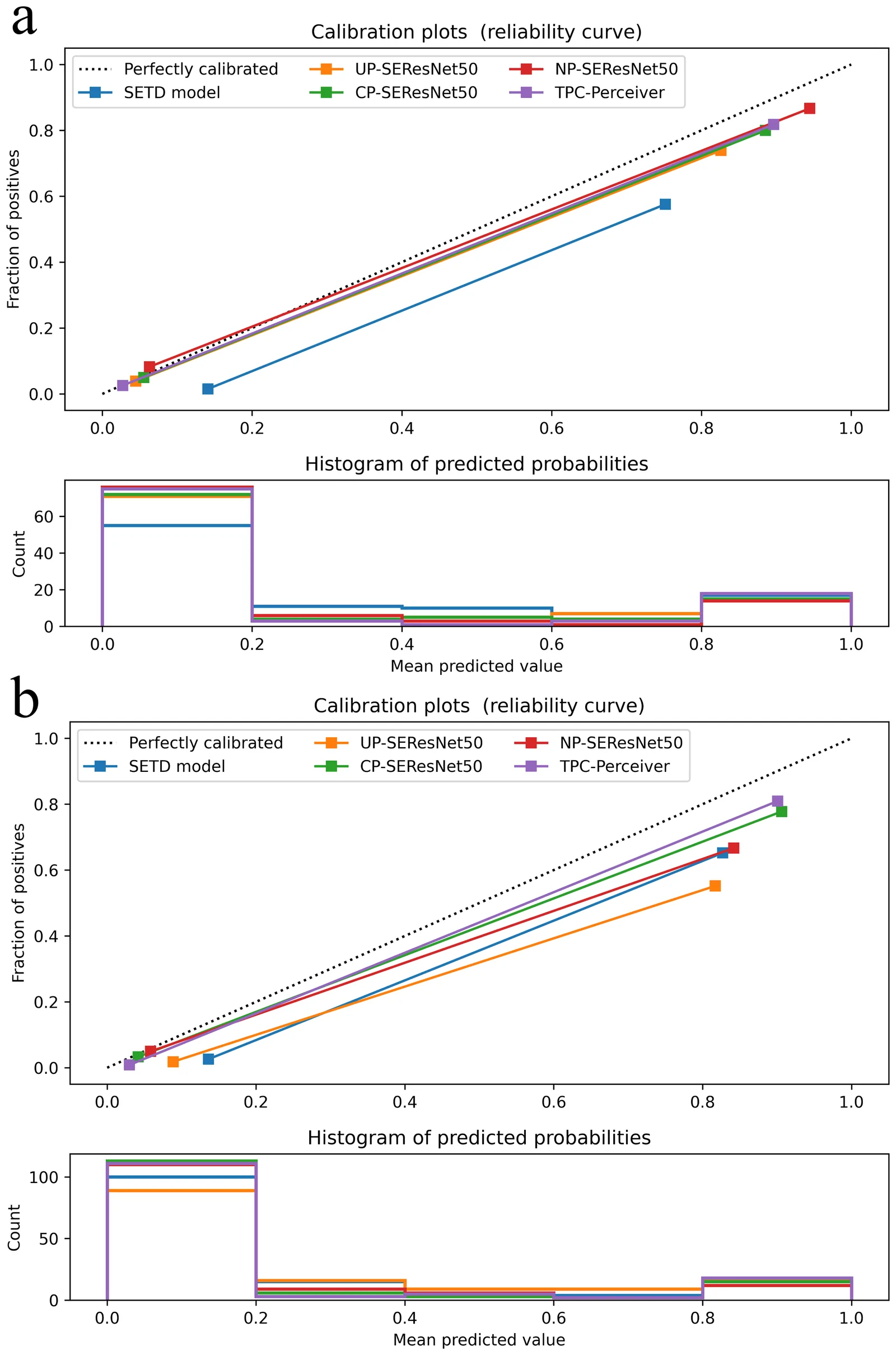
The Calibration curves for each model in validation and testing datasets. **(a)** The Calibration curves in validation datasets, **(b)** The Calibration curves in testing datasets.

**Figure 8:**
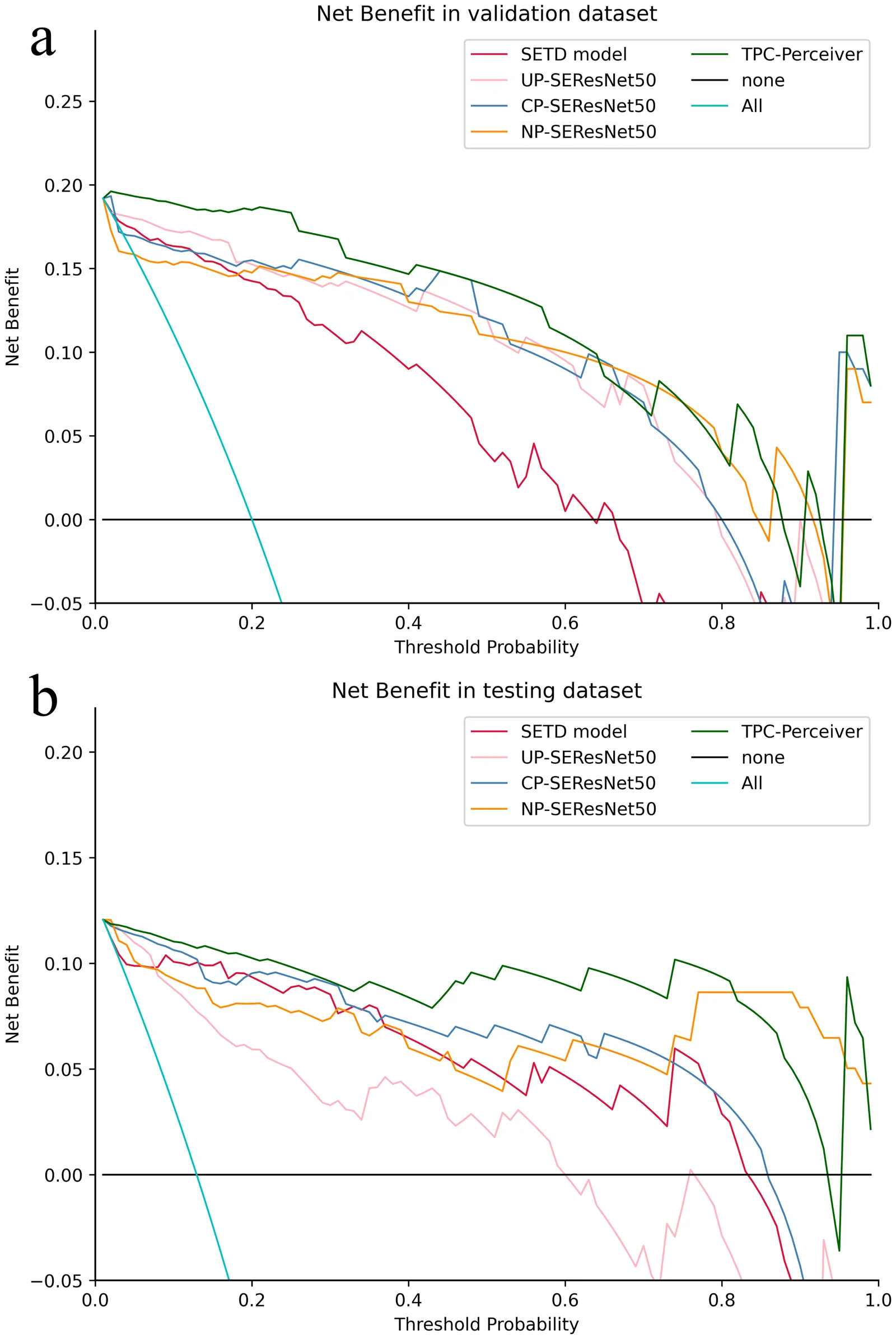
Decision Curve Analysis (DCA) for each model in validation and testing datasets. **(a)** DCA in validation dataset, **(b)** DCA in testing dataset. The y-axis represents the net benefit, while the x-axis represents the corresponding threshold probability. The TPC-Perceiver model is represented by the green line.

## 4 Discussion

In this retrospective multicenter study, we developed and externally evaluated a deep learning framework for malignancy risk stratification of cystic renal lesions (CRLs). The framework combines interactive segmentation with phase-specific feature extraction and TPC-Perceiver fusion of unenhanced, corticomedullary, and nephrographic CT images. In the external test cohort, the TPC-Perceiver achieved an AUC of 0.969, with a sensitivity of 94.4% and a specificity of 96.7% at the fixed probability threshold of 0.5. Exploratory pairwise AUC comparisons favored the TPC-Perceiver over the single-phase SEResNet50 models and the clinical Bosniak v2019 assessment, while its AUC was comparable to that of the previous SETD model. These findings suggest that integrating complementary information from three CT phases may support malignancy risk stratification while maintaining a favorable balance between sensitivity and specificity.

The unenhanced phase provides baseline attenuation for assessing enhancement on postcontrast CT images. Benign cystic lesions generally show little or no enhancement, whereas malignant cystic renal neoplasms may contain enhancing walls, septa, or mural nodules (24, 25). In the present framework, the unenhanced, corticomedullary, and nephrographic phases were processed separately by phase-specific SEResNet50 networks. The Squeeze-and-Excitation modules adaptively recalibrate feature channels, allowing each network to learn phase-specific imaging representations without relying on predefined handcrafted descriptors. These representations were then fused by the TPC-Perceiver through a latent-bottleneck and cross-attention mechanism. This approach provides a structured way to integrate complementary information across the three CT phases.

The ablation analysis compared latent feature fusion with several probability-level combination strategies. All approaches achieved high AUCs, suggesting that information from the three CT phases contributed to discrimination. The TPC-Perceiver achieved the highest AUC in the validation cohort, whereas its external-test AUC was lower than those of the probability-level comparators. At the common fixed threshold of 0.5, no strategy was uniformly superior across accuracy, sensitivity, specificity, and F1-score. These results support further evaluation of Perceiver-based fusion, but its added value over simpler probability-level combinations remains to be established in larger independent cohorts.

Accurate lesion segmentation is important for image-based characterization of complex CRLs. Manual volumetric delineation is labor-intensive and potentially subjective. Fully automated segmentation can also be difficult when lesion boundaries are irregular or poorly defined. In this study, the interactive Swin UNETR combined automated volumetric segmentation with radiologist-guided corrective points. It achieved mean Dice scores of 0.885 and 0.890 in the internal validation and external test cohorts, respectively. Positive and negative interaction points allowed residual under- or over-segmentation to be corrected without complete manual redelineation.

Complex CRLs, particularly Bosniak IIF and III lesions, remain clinically challenging. Management requires a balance between avoiding unnecessary intervention and preventing delayed diagnosis of malignancy (9, 10, 26–29). The high specificity of the TPC-Perceiver suggests that it may help reduce false-positive classifications in selected patients. Decision curve analysis provided additional evidence of its potential clinical utility. The threshold probability represents the predicted malignancy risk at which the expected benefit of intervention is considered to outweigh its potential harms. This threshold may vary with lesion characteristics, comorbidities, renal function, and patient preferences. We therefore evaluated net benefit across a broad range of threshold probabilities rather than at a single clinical cutoff. A false-positive prediction may lead to unnecessary imaging, biopsy, or surgery for a benign lesion. In contrast, a false-negative prediction may delay the diagnosis and treatment of malignancy. In practice, a low predicted risk may support active surveillance in appropriately selected patients. An intermediate risk may prompt further imaging or biopsy when the result is likely to alter management, whereas a high predicted risk may support surgical evaluation. The model should nevertheless be used as an adjunct to multidisciplinary assessment and shared decision-making rather than as a stand-alone treatment decision system.

Histopathology was used as the primary reference standard, and all lesions in the internal validation and external test cohorts were histopathologically confirmed. In the training cohort, 184 of 195 lesions were histopathologically confirmed. The remaining 11 benign lesions without surgical pathology were included based on at least 4 years of CT follow-up showing no definite imaging progression or upgrade in Bosniak classification. Previous longitudinal evidence suggests that progression of Bosniak IIF lesions, when it occurs, is generally detected during the early years of surveillance (30). This provides clinical context for the use of prolonged imaging stability as supportive evidence of benign behavior. Nevertheless, radiological stability is not equivalent to histopathological confirmation and may have introduced some label uncertainty during model training. A sensitivity analysis excluding these 11 lesions was not performed, and their influence on the learned model parameters cannot be completely excluded.

Representative machine-learning and deep-learning studies of cystic renal lesion characterization are summarized in **Supplementary Table 5**. These studies differ in imaging modality, modeling strategy, sample size, reference standard, and validation design. Our previous SETD framework used a 3D ResNet50 encoder and a GRU-based temporal decoder to model multiphase CT information. The present framework additionally incorporates the unenhanced phase and uses the TPC-Perceiver to fuse phase-specific representations. Given the methodological differences among published studies, their reported performance provides useful context but should not be interpreted as a direct head-to-head comparison.

Several limitations should be considered. First, the retrospective design introduces potential selection bias. The external test cohort included only 18 malignant lesions, which limits the precision of the performance estimates. This is particularly relevant for metrics approaching 0% or 100%. No *a priori* sample-size calculation was performed because the cohort size was determined by the number of eligible cases available during the study period. Larger prospective multicenter cohorts with more malignant lesions are needed to obtain more stable estimates.

Second, CT scanner platforms and acquisition parameters differed among the participating institutions. All images underwent the same preprocessing pipeline, but no dedicated statistical harmonization or domain-adaptation procedure was applied. Data from the two external centers were pooled to evaluate performance across heterogeneous imaging conditions. However, this approach may have obscured center-specific or scanner-specific effects. Separate analyses were not performed because further stratification would have resulted in very small malignant subgroups.

Third, the interactive segmentation process requires radiologist input. The effects of interaction-point number, interaction-point location, and operator experience were not systematically evaluated. We also did not conduct a paired multi-operator experiment in which the same lesions were independently assessed by readers with different levels of experience. The influence of interaction strategy and operator variability on segmentation and downstream classification therefore remains uncertain.

Fourth, the comparison with Bosniak v2019 should be interpreted cautiously. The Bosniak categories were retrospectively extracted from finalized clinical radiology reports generated through routine departmental interpretation and senior review. Individual pre-finalization ratings were not separately retained. Inter-reader agreement and reader-specific diagnostic performance could therefore not be evaluated. The reported performance represents the finalized clinical assessments in the present cohorts rather than a system-level estimate of Bosniak v2019 performance across readers and institutions.

Fifth, model interpretability remains limited. The image regions and imaging features contributing to individual predictions were not systematically evaluated using validated class-specific attribution methods. Future studies should incorporate reproducible explainable-AI approaches, such as Grad-CAM or other attribution methods. Radiologist assessment of the resulting explanation maps will also be needed to determine whether model predictions consistently rely on clinically meaningful lesion characteristics.

Finally, the present framework requires complete tri-phase CT examinations and may not be directly applicable to reduced-phase protocols or other imaging pathways. Lesions smaller than 1 cm were excluded, and performance in sub-centimeter CRLs remains unknown. The study also evaluated CT exclusively and did not directly assess MRI-based classification (31–34). Future studies should evaluate the framework prospectively across broader imaging protocols and smaller lesions. Multimodal CT–MRI models incorporating relevant clinical information also warrant investigation.

## 5 Conclusion

In summary, this retrospective multicenter study developed and externally evaluated a deep learning framework combining interactive segmentation with tri-phase CT feature integration for malignancy risk stratification of cystic renal lesions. The TPC-Perceiver achieved favorable discrimination and a balanced sensitivity-specificity profile at the fixed probability threshold of 0.5. Exploratory AUC comparisons favored the TPC-Perceiver over the single-phase models and the clinical Bosniak v2019 assessment, while its AUC was comparable to that of the previous SETD model. These findings support further evaluation of the framework as an adjunct to clinical assessment. Larger prospective multicenter studies are needed before clinical implementation.

## Data Availability

The original contributions presented in the study are included in the article/**Supplementary Material**. Further inquiries can be directed to the corresponding authors.
