# Supplementary material for: Deep learning with interactive segmentation for risk stratification of cystic renal lesions on tri-phase CT: a multicenter study
Source: Front Oncol. 2026 Oct 2;16:1832969. doi: 10.3389/fonc.2026.1832969 (PMC13671972; doi:10.3389/fonc.2026.1832969)
Supplement: Supplementary file 1 [file Supplementaryfile1.pdf]

Supplementary Material

Supplementary Figures and Tables

Supplementary Figure 1. Detailed structure of the interactive SWIN UNETR model

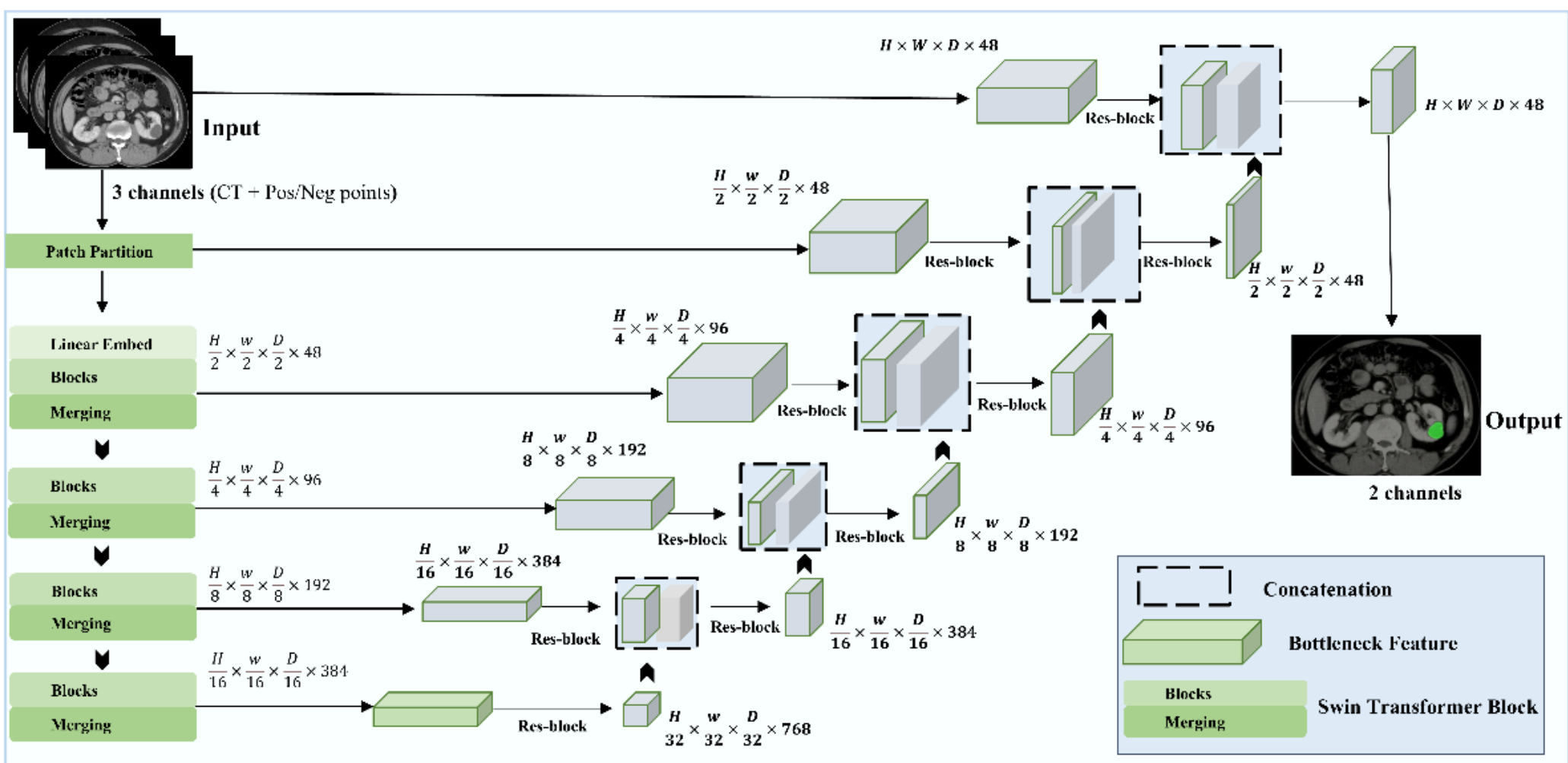

The interactive Swin UNETR network is designed for the interactive segmentation task of medical images. The network adopts a hybrid architecture, utilizing a Swin Transformer-based encoder for hierarchical feature extraction and a CNN-based decoder for precise spatial reconstruction. Specifically, the input to the network has three channels, which consist of the original CT image along with the user-provided positive and negative interaction points. The encoder progressively processes the input through patch partitioning, linear embedding, and successive Swin Transformer blocks with merging layers. In the decoder pathway, the features are upsampled and processed through residual blocks (Res-blocks), utilizing skip connections to concatenate them with the corresponding multi-scale features from the encoder. The final output yields a 2-channel segmentation map.

Supplementary Figure 2. Detailed structure of the TPC-Perceiver model

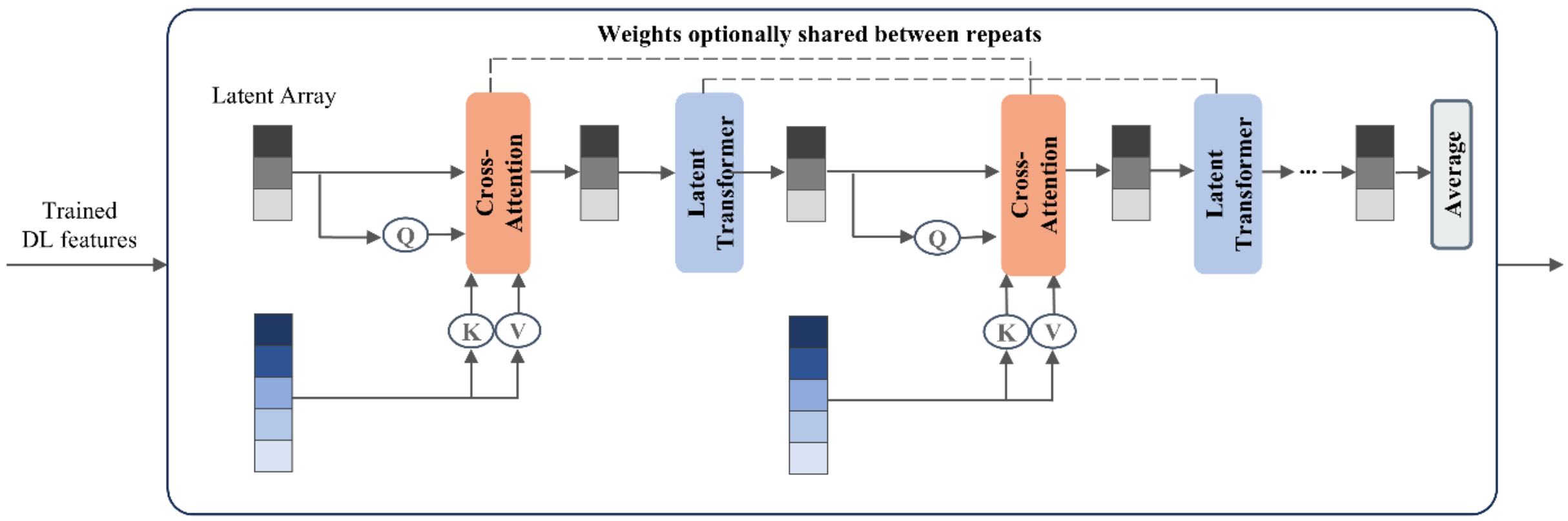

The TPC-Perceiver integrates complementary representations extracted from the unenhanced, corticomedullary, and nephrographic phases. Phase-specific features are provided to a latent-bottleneck module and aggregated through cross-attention. The refined latent representation is then used to generate the predicted probability of malignancy.

Supplementary Figure 3. Confusion matrices for the single-phase deep-learning models at the fixed probability threshold of 0.5 in the validation and test datasets.

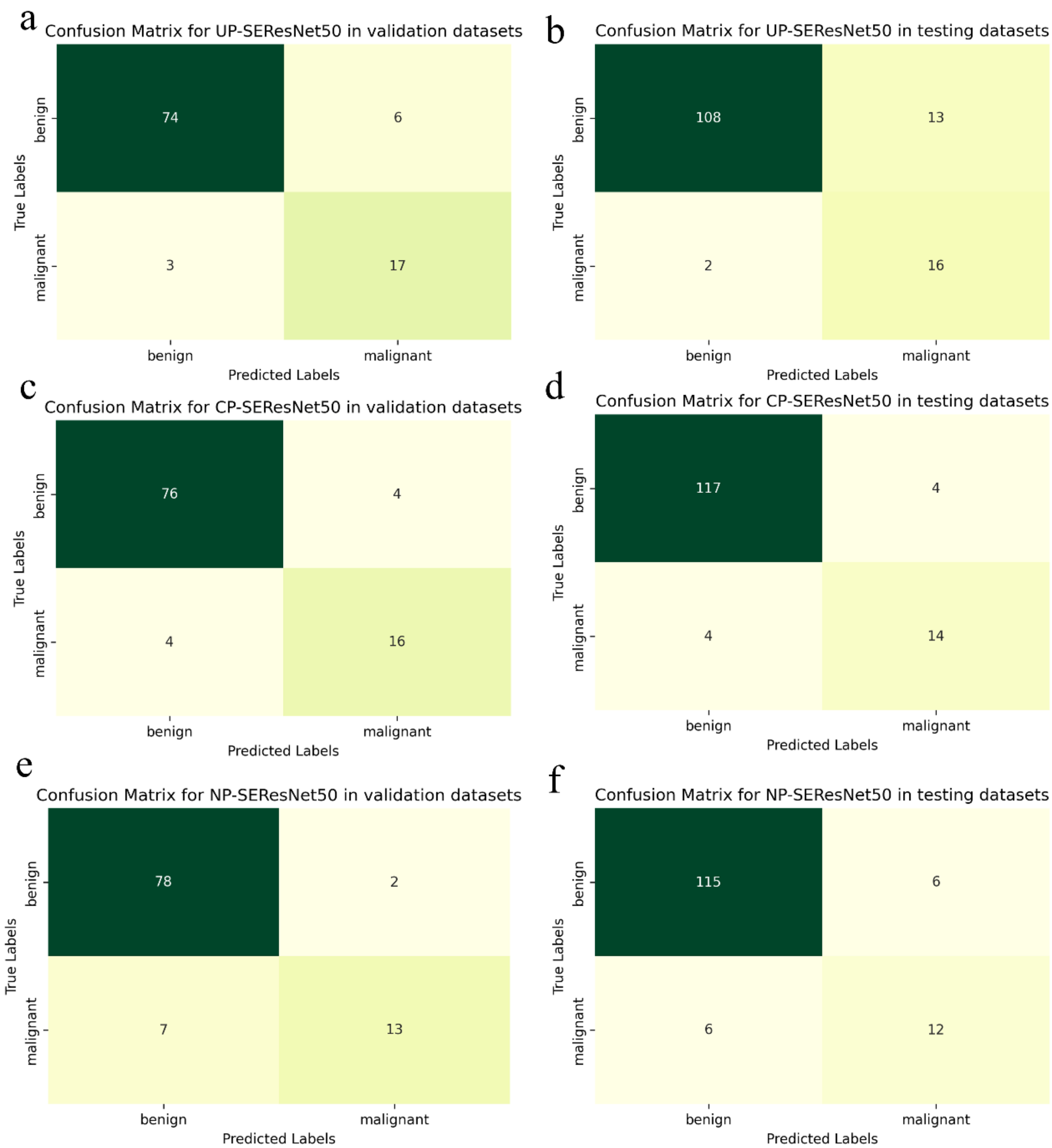

Supplementary Figure 4. Distribution of recorded interactive clicks in the validation and external test cohorts.

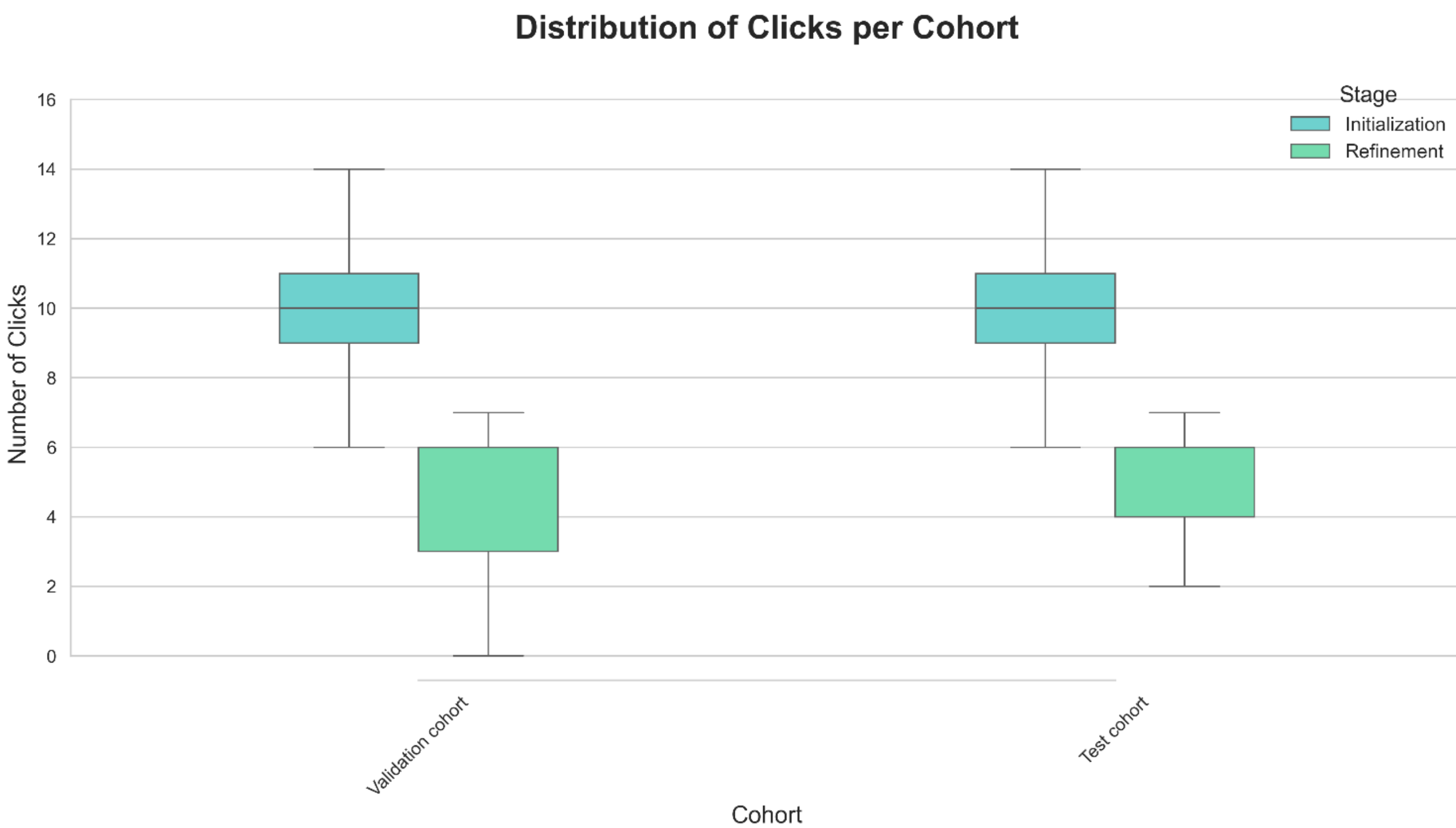

Boxplots show the distributions of manually provided clicks during the initialization and refinement stages of the interactive Swin-UNETR workflow in the validation and external test cohorts. The median number of clicks during initialization was 10 in both cohorts, whereas the median number of corrective clicks during refinement was 6 in both cohorts. Comparable distributions between the two cohorts suggest consistent interaction requirements across datasets.

Supplementary Table 1. Deep learning and radiomics model quality score checklist

Radiomics Quality items	Score
Multi-center study	1
Defined the classification of the model	1
Input from Clinicians for interpretable pipeline development	1
Image protocol quality is well documented and publicly used	2
Description of the hardware used and image reconstruction method	2
Accounted for Preprocessing of the images	1
Imaging at multiple time points	1
Inclusion and exclusion criteria or boundaries of the model defined	1
Use post-processing harmonization to reduce multi-center acquisition variability	1
Cut-off analyses	1
Early stopping method used to avoid the risk of overfitting	1
Comparative analysis between radiomics and deep learning	0
Quality Management System available online with internal audit	4
Adopted bootstrapping resampling method and discrimination plots	2
Adopted bootstrapping resampling method and calibration plots	2
Comparison with previously published radiomics signatures and models	1
Validation is based on three distinct institutes	5
Available detailed evaluation explanations	2
Comparison to 'gold standard'	2
Proved potential clinical utility by decision curve analysis	2
The radiomics model provides the predictions and the clinician's intervention is required for special (out-of-distribution) cases	4
The algorithm and source code are made publicly available.	1
CT acquisition protocols and ROI segmentation methods are reported; patient-level data are not publicly available and may be accessible upon reasonable request	3
Future strategy to update models	1
Total score	42

Supplementary Table 2. Detailed CT imaging scanning protocols in the training cohort and each validation cohort

**Parameter**	**Training cohort**	**Validation cohort**	External validation cohort 1	**External validation cohort 2**
**CT scanner**	128-slice spiral CT (Siemens) or 64-slice spiral CT (GE)	128-slice spiral CT (GE)	128-slice spiral CT (Siemens) or 64-slice spiral CT (GE)	256-slice scanner (Philips)
**CT-tube voltage**	100–120 (kV)	90–120 (kV)	100–120 (kV)	100–120 (kV)
**CT-tube current**	125–300(mAs)	125–350(mAs)	125–350(mAs)	125–360(mAs)
**Scanning matrix**	512*512 pixels	512*512 pixels	512*512 pixels	512*512 pixels
**Body reconstruction kernel**	yes	yes	yes	yes
**Slice thickness**	1 mm to 7 mm	1 mm to 7 mm	1 mm to 7 mm	1 mm to 7 mm
**Contrast volume**	1.5(mL/kg)	1.5(mL/kg)	1.5(mL/kg)	1.5(mL/kg)

Supplementary Table 3. Detailed distribution of Bosniak classification version 2019 and histopathology results in the training cohort, validation cohort and external validation cohorts.

Histopathology analysis & ≥ 4 years of imaging follow-up		Benign results	Malignant results
		**n=140**	**n=55**
**Training cohort**	Bosniak I (n=89)	simple renal cysts (n=89)	(n=0)
	Bosniak II (n=29)	simple renal cysts (n=19)<br>≥ 4 years of imaging follow-up (8)	papillary renal cell carcinoma (n=1)<br>tubulocystic renal cell carcinoma (n=1)
	Bosniak IIF (n=17)	simple renal cysts (n=7)<br>cystic nephroma (n=1)<br>renal angiomyolipoma (n=2)	tubulocystic renal cell carcinoma (n=2), papillary renal cell carcinoma (n=1)<br>clear cell renal cell carcinoma (n=3)<br>multilocular cystic renal neoplasm of low malignant potential (n=1)
	Bosniak III (n=20)	simple renal cysts (n=5)<br>cystic nephroma (n=1)<br>≥ 4 years of imaging follow-up (2)<br>renal angiomyolipoma (n=1)	clear cell renal cell carcinoma (n=6), papillary renal cell carcinoma (n=1)<br>chromophobe renal cell carcinoma (n=1)<br>multilocular cystic renal neoplasm of low malignant potential (n=2)<br>tubulocystic renal cell carcinoma (n=1)
	Bosniak IV (n=40)	renal angiomyolipoma (n=2)<br>cystic nephroma (n=2)<br>≥ 4 years of imaging follow-up (n=1)	unclassified renal cell carcinoma (n=5), renal collecting duct carcinoma (n=1)<br>clear cell renal cell carcinoma (n=21)<br>papillary renal cell carcinoma (n=5), chromophobe renal cell carcinoma (n=2)<br>multilocular cystic renal neoplasm of low malignant potential (n=1)
**Histopathology analysis**		**n=80**	**n=20**
**Validation cohort**	Bosniak I (n=35)	simple renal cysts (n=35)	(n=0)
	Bosniak II (n=15)	simple renal cysts (n=15)	(n=0)
	Bosniak IIF (n=24)	simple renal cysts (n=19)<br>mixed epithelial and stromal tumor (n=1)	clear cell renal cell carcinoma (n=2), papillary renal cell carcinoma (n=1)<br>multilocular cystic renal neoplasm of low malignant potential (n=1)
	Bosniak III (n=13)	simple renal cysts (n=5)<br>mixed epithelial and stromal tumor (n=1)	clear cell renal cell carcinoma (n=7)
	Bosniak IV (n=13)	simple renal cysts (n=1)<br>cystic nephroma (n=1)<br>renal angiomyolipoma (n=1)	clear cell renal cell carcinoma (n=7)<br>unclassified renal cell carcinoma (n=1)<br>multilocular cystic renal neoplasm of low malignant potential (n=1)

		renal oncocytoma (n=1)	
**Histopathology analysis**		**n=121**	**n=18**
External validation cohort 1 and 2	Bosniak I (n=78)	simple renal cysts (n=78)	(n=0)
	Bosniak II (n=15)	simple renal cysts (n=15)	(n=0)
	Bosniak IIF (n=24)	simple renal cysts (n=21)	multilocular cystic renal neoplasm of low malignant potential (n=3)
	Bosniak III (n=12)	simple renal cysts (n=4)	clear cell renal cell carcinoma (n=4), renal collecting duct carcinoma (n=2)<br>papillary renal cell carcinoma (n=1)<br>unclassified renal cell carcinoma (n=1)
	Bosniak IV (n=10)	simple renal cysts (n=3)	clear cell renal cell carcinoma (n=4), unclassified renal cell carcinoma (n=2)<br>multilocular cystic renal neoplasm of low malignant potential (n=1)

Supplementary Table 4: Ablation analysis comparing the TPC-Perceiver with simpler multi-phase fusion strategies.

Fusion strategy	Validation AUC	Test AUC	Test ACC	Test SEN	Test SPE
Equal-probability averaging	0.975	0.983	95.0%	88.9%	95.9%
Logistic-regression stacking	0.974	0.983	97.1%	83.3%	99.2%
Engineered probability features + logistic regression	0.975	0.983	95.7%	72.2%	99.2%
Confidence-weighted probability pooling	0.979	0.983	92.1%	94.4%	91.7%
TPC-Perceiver (Proposed)	0.984	0.969	96.4%	94.4%	96.7%

The four comparators combined phase-specific prediction probabilities produced by the same SEResNet50 backbones, whereas the TPC-Perceiver fused latent feature representations. In the external test cohort, the AUC was 0.969 for the TPC-Perceiver and 0.983 for each probability-level comparator. Formal pairwise significance results are not reported because a valid paired comparison requires verified patient-level alignment across prediction files. At the common fixed probability threshold of 0.5, the methods showed different sensitivity-specificity trade-offs. The TPC-Perceiver achieved an accuracy of 96.4%, sensitivity of 94.4%, specificity of 96.7%, and F1-score of 0.872. Across the probability-level comparators, accuracy ranged from 92.1% to 97.1%, sensitivity from 72.2% to 94.4%, specificity from 91.7% to 99.2%, and F1-score from 0.756 to 0.882. No method was uniformly superior across all threshold-dependent metrics. AUC, area under the receiver operating characteristic curve; ACC, accuracy; SEN, sensitivity; SPE, specificity.

Supplementary Table 5. Comparison of representative published machine-learning and deep-learning studies for cystic renal lesion characterization

Study	Imaging modality / method	Study design / sample size	Reference standard	Validation strategy	Reported performance
Miskin et al. [19]	CT / machine learning	Retrospective; n=257	Bosniak v2019 assessment	Cross-validation	AUC 0.90; SEN 0.63; SPE 0.93
Dana et al. [21]	Contrast-enhanced CT / machine learning	Retrospective, two-center; n=199	Histopathology & long-term imaging follow-up	Independent test cohort	AUC 0.96; BACC 0.94
Kang et al. [33]	MRI / machine learning	Retrospective, multicenter; n=326	Histopathology	Independent test cohort	AUC 0.907; ACC 0.833
He et al. [4]	Contrast-enhanced CT / Deep learning	Retrospective, multicenter; n=715	Histopathology & long-term imaging follow-up	Multicenter external test cohorts	AUC 0.998; SEN 1.000; SPE 0.972
Present study	Tri-phase CT / Deep learning	Retrospective, multicenter; n=434	Histopathology (validation/test) & long-term imaging follow-up	Multicenter external test cohorts	AUC 0.969; SEN 0.944; SPE 0.967

AUC, area under the receiver operating characteristic curve; SEN, sensitivity; SPE, specificity; ACC, accuracy; BACC, balanced accuracy. Reported performance corresponds to the validation or test setting reported in each study. Direct numerical comparisons should be interpreted cautiously because of differences in study populations, imaging modalities, reference standards, and validation strategies.

CLEAR Checklist v1.0

Note: Use the checklist in conjunction with the main text for clarification of all items.
Yes, details provided; No, details not provided; n/e, not essential; n/a, not applicable; Page, page number

Section	No.	Item	Yes	No	n/a	Page
**Title**						
	1	Relevant title, specifying the radiomic methodology	☑	☐	☐	1
**Abstract**						
	2	Structured summary with relevant information	☑	☐	☐	1-2
**Keywords**						
	3	Relevant keywords for radiomics	☑	☐	☐	2
**Introduction**						
	4	Scientific or clinical background	☑	☐	☐	2-3
	5	Rationale for using a radiomic approach	☑	☐	☐	4
	6	Study objective(s)	☑	☐	☐	4
**Method**						
Study design	7	Adherence to guidelines or checklists (e.g., CLEAR checklist)	☑	☐	☐	5
	8	Ethical details (e.g., approval, consent, data protection)	☑	☐	☐	5
	9	Sample size calculation	☐	☐	☑	
	10	Study nature (e.g., retrospective, prospective)	☑	☐	☐	5
	11	Eligibility criteria	☑	☐	☐	5
	12	Flowchart for technical pipeline	☑	☐	☐	5
Data	13	Data source (e.g., private, public)	☑	☐	☐	5
	14	Data overlap	☑	☐	☐	5
	15	Data split methodology	☑	☐	☐	5
	16	Imaging protocol (i.e., image acquisition and processing)	☑	☐	☐	6
	17	Definition of non-radiomic predictor variables	☐	☐	☑	
	18	Definition of the reference standard (i.e., outcome variable)	☑	☐	☐	6
Segmentation	19	Segmentation strategy	☑	☐	☐	6
	20	Details of operators performing segmentation	☑	☐	☐	6
Pre-processing	21	Image pre-processing details	☑	☐	☐	6
	22	Resampling method and its parameters	☑	☐	☐	6
	23	Discretization method and its parameters	☑	☐	☐	6
	24	Image types (e.g., original, filtered, transformed)	☑	☐	☐	6
Feature extraction	25	Feature extraction method	☑	☐	☐	7
	26	Feature classes	☑	☐	☐	7
	27	Number of features	☑	☐	☐	7
	28	Default configuration statement for remaining parameters	☑	☐	☐	7
Data preparation	29	Handling of missing data	☐	☐	☑	
	30	Details of class imbalance	☑	☐	☐	6
	31	Details of segmentation reliability analysis	☑	☐	☐	7
	32	Feature scaling details (e.g., normalization, standardization)	☑	☐	☐	7
	33	Dimension reduction details	☑	☐	☐	7
Modeling	34	Algorithm details	☑	☐	☐	8
	35	Training and tuning details	☑	☐	☐	8
	36	Handling of confounders	☑	☐	☐	8
	37	Model selection strategy	☑	☐	☐	8
Evaluation	38	Testing technique (e.g., internal, external)	☑	☐	☐	8-9
	39	Performance metrics and rationale for choosing	☑	☐	☐	8-9
	40	Uncertainty evaluation and measures (e.g., confidence intervals)	☑	☐	☐	8-9
	41	Statistical performance comparison (e.g., DeLong's test)	☑	☐	☐	8-9
	42	Comparison with non-radiomic and combined methods	☑	☐	☐	8-9
	43	Interpretability and explainability methods	☐	☐	☑	
**Results**						
	44	Baseline demographic and clinical characteristics	☑	☐	☐	9
	45	Flowchart for eligibility criteria	☑	☐	☐	9
	46	Feature statistics (e.g., reproducibility, feature selection)	☑	☐	☐	10
	47	Model performance evaluation	☑	☐	☐	10
	48	Comparison with non-radiomic and combined approaches	☑	☐	☐	11
**Discussion**						
	49	Overview of important findings	☑	☐	☐	12
	50	Previous works with differences from the current study	☑	☐	☐	13
	51	Practical implications	☑	☐	☐	14
	52	Strengths and limitations (e.g., bias and generalizability issues)	☑	☐	☐	15

Section	No.	Item	Yes	No	n/a	Page
**Open Science**						
Data availability	53	Sharing images along with segmentation data [n/e]	☐	☐	☑	
	54	Sharing radiomic feature data	☑	☐	☐	9
Code availability	55	Sharing pre-processing scripts or settings	☑	☐	☐	9
	56	Sharing source code for modeling	☑	☐	☐	9
Model availability	57	Sharing final model files	☑	☐	☐	9
	58	Sharing a ready-to-use system [n/e]	☑	☐	☐	9

Kocak B, Baessler B, Bakas S, Cuocolo R, Fedorov A, Maier-Hein L, Mercaldo N, Müller H, Orlhac F, Pinto Dos Santos D, Stanzione A, Ugga L, Zwanenburg A. CheckList for EvaluAtion of Radiomics research (CLEAR): a step-by-step reporting guideline for authors and reviewers endorsed by ESR and EuSoMII. Insights Imaging. 2023 May 4;14(1):75. doi: 10.1186/s13244-023-01415-8
